# Commentary on the fundamentals and development of artificial intelligence models in the life sciences and best research practices

**DOI:** 10.1007/s10822-026-00870-x

**Published:** 2026-07-03

**Authors:** Jürgen Bajorath

**Affiliations:** 1 Department of Life Science Informatics, B-IT, Rheinische Friedrich-Wilhelms-Universität, Bonn, Germany; 2 Lamarr Institute for Machine Learning and Artificial Intelligence, Rheinische Friedrich-Wilhelms-Universität, Bonn, Germany; 3 LIMES Program Unit Chemical Biology and Medicinal Chemistry, Rheinische Friedrich-Wilhelms-Universität, Friedrich-Hirzebruch-Allee 6, Bonn, D-53115 Germany

**Keywords:** Artificial intelligence, Machine learning, Life and natural sciences, Model development and evaluation, Best practices for interdisciplinary research

## Abstract

Machine learning has a long history in the life and natural sciences. In the age of artificial intelligence (AI), both the opportunities and challenges associated with predictive and generative modeling are increasing. To exert significant influence on experimental programs in interdisciplinary research, AI models and their predictions must be demystified as far as possible, and misconceptions regarding what highly complex models can and cannot do must be avoided. Furthermore, it is necessary to evaluate best practices for the use of AI in life science research including drug discovery. This contribution discusses the evolution of AI models in the life and natural sciences, as well as current developments and remaining challenges in this rapidly evolving field. It is dedicated to the memory of Terry R. Stouch, a valued colleague and dear friend whom I had the privilege of knowing for more than 30 years and who left us far too soon.

## Background and context

Machine learning (ML) methods have been applied in the life and natural sciences since the 1980s. In computer science, the deep learning (DL) era began about 15 years ago when significant progress was made using deep neural networks (DNNs) in areas such as natural language processing (NLP) [1] and computer vision [2]. The success of DNNs in these and other areas stemmed primarily from the ability of these models to learn relevant features or patterns from available data without the need for predefined data structures; a key difference compared to other ML methods. In NLP, recursive DNNs and especially transformer networks [3] capture information hierarchies iteratively, ranging from individual words and sentences to contextual relationships spanning longer passages of text. Transformers were introduced in 2017 and represent the foundation of today’s large language models (LLMs) [3]. Digital images consist of a large number of pixel values from which architectures such as convolutional DNNs automatically extract and learn relevant image features and structures. A key characteristic of DNNs originating from NLP or computer vision is that these networks can be used for both predictive and generative modeling, as further discussed below. Over the past decade, DL models have been increasingly adapted in the life and natural sciences and shaped computer-aided design in different areas including pharmaceutical research and material science.

## Data limitations and deep learning

The data distribution in the life and natural sciences is highly heterogeneous. Some disciplines such as particle physics or genomics are data-rich, whereas others including medicinal chemistry are comparatively data-poor. However, in chemistry, well-defined molecular representations are available. The data situation here is thus completely different from, for instance, image processing, which involves vast amounts of unstructured data from which representations must be learned. Comparatively small volumes of well-structured data often do not allow DL to leverage its strengths. Consequently, under such conditions, predictive DNNs are not necessarily superior to simpler ML models, which has often been shown in cheminformatics and medicinal chemistry applications. Clearly, relative predictive performance of ML models including DNNs generally depends on the specific applications and data characteristics.

## New opportunities

However, DNNs have opened up novel possibilities for generative modeling to which other ML methods were not applicable [4]. For example, in molecular design, de novo generation of small molecules with an unprecedented degree of chemical diversity has become feasible as well as the generation of compounds with a wide range of biological activities [5]. Much progress has also been made, for instance, in synthesis planning and design [6]. In recent years, transformer and diffusion models [7] have emerged as the leading DNN architectures for generative modeling in biology, chemistry, and drug design. For example, transformers are widely used as chemical or biological language models [8]. Furthermore, diffusion models [7], which were originally used primarily for image generation, are now at the forefront of protein design and are increasingly used for de novo structure-based compound design.

## Explainable artificial intelligence

Despite these positive developments, some significant challenges remain. One of these is the “black box” nature of most ML and all DL models. In this context, the term black box refers to the fact that the way these models arrive at their predictions cannot be traced or understood in terms of human comprehension. This is not only intellectually unsatisfactory but also has consequences for the significance and impact of predictive and generative modeling in the life and natural sciences. In interdisciplinary research fields such as drug discovery, the black box of AI models limits their practical impact on experimental programs. Experimental investigators have little to no motivation to plan their work based on predictions they cannot understand, rightly so. In principle, this issue affects all research areas involving potential intersections between computation and experiment, and the problem is not new. Since the early days of ML in the natural sciences, black box computing has caused a discrepancy between predictions and experimental applications, and this gap is widening in the AI era. Despite the rapid development of increasingly complex AI models, their current impact on experimental programs remains limited. However, if predictions fail to ultimately lead to new experiments and improved results, interest in AI models in interdisciplinary research will wane, and available resources will dwindle. Consequently, the field of Explainable AI (XAI) [9] is attracting growing interest across various scientific disciplines. XAI focuses on the development and application of methods to better understand the inner workings of AI models and explain predictions. Such methods are crucial both for rationalizing black box predictions and for formulating experimentally testable hypotheses. When predictions cannot be explained, models are frequently misinterpreted and erroneously evaluated. If results are based on incorrect assumptions, models turn into “Clever Hans” predictors [10], meaning that plausible results arise from other than assumed (but unproven) reasons including artifacts; a major caveat for practical applications of AI models.

## Correlations versus causality

When predictions can be explained computationally and interpreted in a way that is understandable to humans, causal relationships are often examined. Current AI models detect correlations, that is, they rely on identifying statistically significant associations between data features and potential prediction outcomes. Importantly, however, statistically significant correlations do not prove causal relationships. Therefore, the field of Causal ML [11] aims to go beyond the detection of correlations and explore cause-and-effect relationships that might be inherent in the data. However, when predicting the outcome of natural processes (such as a chemical reaction or a biological test), causal relationships are defined differently: in such cases, causality exists if the features prioritized by a model for a specific prediction are directly responsible for the corresponding outcome of the natural process. Thus, if meaningful hypotheses regarding feature relevance can be formulated, experimental hypothesis testing is typically required.

## Model evolution

**Fig. 1:**
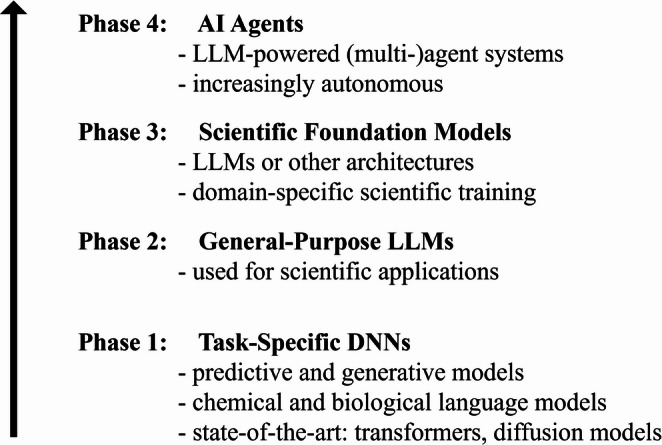
AI models used for scientific applications. Beginning with task-specific DNNs, different types of AI models have been increasingly used for scientific applications (as indicated by the arrow on the left). From phase 1–4, the complexity of AI models is increasing

For applications in the life and natural sciences, different types of AI models have been increasingly used, as summarized in Fig. 1.

While task-specific predictive and generative DNNs continue to be widely used, LLMs are also employed for scientific applications, even though they are not specifically trained for science. A major attraction is their interactive use in natural language, different from task-specific models. However, scientific applications of LLMs are frequently reported without rigorous evaluation or controls. LLMs or other DNN architectures may undergo domain-specific scientific training to generate scientific foundation models, which have been introduced for different disciplines across the life and natural science including drug discovery [12]. Moreover, LLMs with or without additional scientific training are increasingly being used as a central component to power AI agent systems [13]. In its simplest form, an AI agent represents a model with a specific function. Scientific foundation models can also act as agents. In multi-agent systems, the central LLM controls the actions and sequential deployment of individual agents that perform specific tasks. For instance, specifically trained agents might search databases, analyze data, generate scientific hypotheses, design sequences of computational operations to test hypotheses, generate code, execute tasks for hypotheses testing, or review the output of other agents. While many agent systems are still in their early stages, these systems are designed to become increasingly autonomous in conducting research.

A major challenge in the scientific use of LLMs, regardless of whether operated as individual models or as a central component of agent systems, is their tendency toward hallucinations, that is, they can generate seemingly plausible results that are not based on facts or empirical evidence. Scientific hallucinations might propagate through agent systems and further complicate the issue. LLM-based scientific foundation models should in principle reduce and better control scientific hallucinations. However, detailed comparisons of LLMs and scientific foundation models are currently lacking. Conducting such comparisons will be essential for critically evaluating and further developing these systems.

The currently most advanced scientific AI agents include LLM-based hierarchical multi-agent architectures such as the Virtual Lab of AI Agents [14] or a single LLM-based agent that uses foundation models with different roles, as exemplified by the Virtual AI Scientist [15]. In the hierarchical Virtual Lab of AI Agents, individual AI agents play different scientific roles and communicate with each other like a research team. Scientist agents are supervised by principal investigator agents and their output is reviewed by critic agents. Iterative communications across the agent hierarchy and refinements should improve the team’s performance. The Virtual AI Scientist is designed for end-to-end research. Its internal roles include research planning, code generation, task execution, analysis of results, preparation of manuscripts, and review. The development of AI agents is a highly dynamic field, and new systems are being introduced at an increasing pace. Of note, given the complexity of agent systems, XAI methods for explaining individual predictions are of little practical relevance in this context. Instead, these systems face the challenge of meeting the standards of Trustworthy AI [16], that is, they must be technically reliable, transparent, and secure, and must ensure data security and ethical standards in the conduct of research.

## Best practices

The requirements for emerging agentic systems are directly linked to the question of which criteria should generally guide the use of AI in research practice. In 2021, Terry Stouch and colleagues published a feature article discussing best practices for AI in life science research [17]. This article was written from the industrial perspective of the Pistoia Alliance [18], but is of general relevance for the scientific use of AI. It appeared about 18 months before AI reached the next level in society and science with the introduction of ChatGPT, making it particularly interesting to view this discussion through today’s AI-centric perspective.

Specifically, the authors proposed 11 best practices for the use of AI in the life sciences and drug discovery and assigned them to three categories (quality data, quality methods, and quality organization) [17], as reported in Table 1.

**Table 1:** Best practices proposed for the use of AI in life science research

Quality data
**1.** Invest in the knowledge domain needed to properly analyze data in highly specialized area
**2.** Use only quality data for training of models
**3.** Use a standard methodology such as FAIR principles for data life-cycle planning

Quality models
**4.** Publish model code and training and test data, sufficient for reproducing the results
**5.** Use a model management system
**6.** Select ML models that fit the specific use case or problem class

Quality organization
**7.** Set reasonable expectations
**8.** Add the skill sets relevant to AI and ML to the company’s leadership
**9.**. Combine AI models with human insights
**10.**. Fail fast
**11.** Create an AI Center of Excellence

Best practices 1–11 are reported according to [17]. FAIR stands for findable, accessible, interoperable, and reusable.

As proposed, the best practices focus largely (but not exclusively) on ML. The first three best practices forming the quality data category generally apply to data requirements for ML, regardless of the methods that are used. For the use of life science data, an understanding of experimental sources and variances is crucial. The quality model category begins with best practice 4 that is imperative to ensure reproducibility of ML results and promote open science. The following best practice, which calls for model management systems taking model life cycles into account, has further gained in importance over the past five years. This is the case because new AI models -and new types of models- are introduced at a high frequency. In fact, at this stage, a well-organized public repository for published AI models would significantly support model development, evaluation, and the pursuit of reproducibility of reported results. Best practice 6, too, has become more important than ever. As AI model complexity further increases, task-specific models must be critically evaluated and compared to prioritize the simplest models yielding desired accuracy for given prediction tasks. This avoids methodological overkill, which is disruptive for meaningful practical applications of AI models, as well as a flawed scientific assessment of alternative methods. The remaining best practices fall into the quality organization category and primarily focus on the use of AI in the pharmaceutical and biotechnology industry. However, similar to best practices 5 and 6, as discussed above, best practices 7, 9, and 10 have become increasingly important for applied AI research over the past years. Setting reasonable expectations (practice 7) is of prime importance for the field. One should add here that the same applies to avoiding misunderstandings of AI technologies and of what AI models can -and cannot- do. In addition, combining AI models with human insights (9) is essential for model evaluation and a pre-requisite for model interpretation and the exploration of causal relationships. This is also directly linked to the “fail fast” paradigm (10) that requires formulating and testing of hypotheses to evaluate model relevance for predicting natural processes. It should be added here that adherence to best practices 9 and 10 also supports the general impact of AI models and their predictions on interdisciplinary research, as discussed above. This has been critical for ML in the life and natural sciences since its inception and continues into the age of AI. Following the introduction and increasing adoption of LLMs, beginning soon after the proposal by Terry and colleagues was published, additional best practices can be formulated. For instance, for LLMs with or without scientific training, rigorous evaluation and validation studies are required. Given the specific features and interactive use of these models, there is a need for new validation concepts beyond conventional benchmarking. In addition, for LLMs used in science, whether as independent models or as the “brain” of AI agent systems, it is necessary to examine their ability to generalize beyond initial case studies. Furthermore, agent architectures must ultimately meet the standards of Trustworthy AI.

In conclusion, the best practices for the use of AI in life science research proposed by Terry and his colleagues have not only proven their worth in this fast-paced field with its numerous recent developments, but have in a number of cases even gained in importance over time.

## Data Availability

No datasets were generated or analysed during the current study.
